# 

**Published:** 1892-11

**Authors:** 


### NOTICE, DOCTORS !

Since the editorial entitled "Some Legislation that We Need" was written and read before the Atlanta Society of Medicine a committee has been appointed by the Society to prepare and submit a bill to the General Assembly looking to the establishment of a Board of Examiners for the State. This committee consists of Dr. Jas. B. Baird, Chairman; Dr. A. W. Calhoun, Dr. W. P. Nicolson, Dr. F. W. McRae and Dr. J. S. Todd. The doctors in the State who will read this notice and who are in sympathy with this movement are requested to communicate with their representatives and lend their assistance to this good cause.

### PAMPHLETS RECEIVED.

Asepsis and Antisepsis as Applied in the Lying-in Chamber. By W. W. Potter, M. D., Buffalo, N. Y. Reprint.

Pelvic Inflammation in Women; a Pathological Study. By W. W. Potter, M. D., Buffalo, N. Y. Reprint.

Report on Abdominal and Pelvic Surgery, Including Thirty-Two Successful Cases of Laparotomy. By Wm. H. Wathen, M. D., Louisville, Ky. Reprint.

Tuberculin and the Living Cell. By Charles Denison, M. D Denver. Reprint.

